# The Impact of Sepsis on the Outcomes of COPD Patients: A Population-Based Cohort Study

**DOI:** 10.3390/jcm7110393

**Published:** 2018-10-27

**Authors:** Cheng-Hsin Chen, Chih-Cheng Lai, Ya-Hui Wang, Cheng-Yi Wang, Hao-Chien Wang, Chong-Jen Yu, Likwang Chen, Taiwan Clinical Trial Consortium for Respiratory Diseases (TCORE)

**Affiliations:** 1 Department of Internal Medicine, Cardinal Tien Hospital and School of Medicine, College of Medicine, Fu-Jen Catholic University, No.362, Zhongzheng Rd., Xindian Dist., New Taipei City 23148, Taiwan; b8603227@gmail.com; 2 Department of Intensive Care Medicine, Chi Mei Medical Center, Liouying 73657, Taiwan; dtmed141@gmail.com; 3 Medical Research Center, Cardinal Tien Hospital and School of Medicine, College of Medicine, Fu Jen Catholic University, New Taipei City 23148, Taiwan; likwang.chen@gmail.com; 4 Department of Internal Medicine, National Taiwan University Hospital and College of Medicine, National Taiwan University, Taipei 10048, Taiwan; jefferycjyu@ntu.edu.tw; 5 Institute of Population Health Sciences, National Health Research Institutes, Zhunan 35053, Taiwan; likwang.chen@gmail.com

**Keywords:** Chronic obstructive pulmonary disease (COPD), sepsis, exacerbation, pneumonia, mortality

## Abstract

This study aims to identify the impact of new-onset sepsis in patients with chronic obstructive pulmonary disease (COPD) including the effects on acute exacerbations, pneumonia and mortality. Using the National Health Insurance Research Database of Taiwan, all patients with COPD older than 40 years between 1988 and 2010 were recruited. After propensity score matching, each of the 8774 COPD patients with and without sepsis were identified to have similar characteristics. The primary outcome was severe exacerbations of COPD, with a severe exacerbation being defined as a patient requiring hospital admission or an emergency department visit due to COPD. The secondary outcomes were pneumonia, serious pneumonia, and all-cause mortality. The post-index overall cumulative incidence rates of total acute exacerbations were 11.2/person-years in the sepsis group and 6.2/person-years in the non-sepsis group (adjusted hazard ratio (HR) = 1.38, 95% confidence interval (CI), 1.38–1.40). The sepsis group also had higher risks of severe exacerbations (adjusted HR = 2.05, 95% CI, 2.02–2.08), severe exacerbations requiring hospitalization (adjusted HR = 2.30, 95% CI, 2.24–2.36), and severe exacerbations leading to an emergency room visit (adjusted HR = 1.91, 95% CI, 1.87–1.94). Regarding the effect on secondary outcomes, the sepsis group had higher risks of mortality (incidence rate: 23.7/person-years vs. 11.34/person-years, adjusted HR = 2.27, 95% CI, 2.14–2.41), pneumonia (incidence rate: 26.41 per person-days vs. 10.34 per person-days, adjusted HR = 2.70, 95% CI, 2.5–2.91), and serious pneumonia (incidence rate: 5.84 per person-days vs. 1.98 per person-days, adjusted HR = 2.89, 95% CI, 2.5–3.33) compared with the non-sepsis group. Sepsis survivors among patients with COPD had a higher risk of severe exacerbations, pneumonia, serious pneumonia, and mortality compared to patients with COPD without sepsis.

## 1. Introduction

Chronic obstructive pulmonary disease (COPD) is a common disease characterized by persistent airflow limitation and associated respiratory symptoms. It is currently the fourth leading cause of death worldwide [1]. Globally, the prevalence of COPD is expected to increase due to a prolonged lifespan and increased exposure to the risk factors of COPD. An exacerbation of COPD is a critical acute event characterized by the worsening of respiratory symptoms, and it is associated with an accelerating decline in lung function. This has a negative effect on the quality of life and increases the rates of hospitalization and mortality [2]. Several factors may induce acute exacerbations in patients with COPD, including air pollution, poor compliance, and most commonly, respiratory tract infections [3,4]. The early identification of the predisposing factors is an important issue in the prevention of acute exacerbations and to improve outcomes in COPD.

Sepsis is a life-threatening condition defined by organ dysfunction caused by a dysregulated host response to infection [5]. Patients with COPD have been reported to be at a higher risk of developing sepsis due to the use of corticosteroids, underlying comorbidities, and possibly impaired barrier function [6,7]. However, the role of sepsis in increasing the risk of acute exacerbations and the effect on the outcomes in COPD has yet to be well defined.

The National Health Insurance (NHI) program in Taiwan was initiated in March 1995. It is a public health insurance system for the general population in Taiwan, and it provides medical care for up to 99% of the 23 million residents of Taiwan [8]. In this study, the National Health Insurance Research Database (NHIRD) of Taiwan was used to identify the impact of new-onset sepsis in patients with COPD including the effects on acute exacerbations, pneumonia and mortality.

## 2. Methods and Materials

### 2.1. Study Design and Patient Selection

This study was conducted using the NHIRD, which is a database constructed by the National Health Research Institutes (NHRI) and includes comprehensive medical care records of more than 97% of the hospitals and clinics in Taiwan [9]. All claims data of ambulatory care records, outpatient visits, prescriptions, inpatient care records, registration files, and disease and vital status data were retrieved from the NHIRD. Ethical approval was obtained from the Institutional Review Board of the Cardinal Tien Hospital (Number: CTH-106-3-5-060).

All patients with COPD older than 40 years between 2000 and 2010 were recruited for this study. The diagnosis of COPD was defined when the patients had an International Classification of Diseases, Ninth Revision, Clinical Modification (ICD-9-CM) diagnostic code for COPD (491, 492, or 496) in at least one hospital admission or three outpatient visits. Although the diagnosis of COPD is based on the clinical signs/symptoms and the findings of spirometry, these data are not available from NHIRD database. Therefore, like other researches based on health claims databases, we used ICD-9-CM codes of COPD and spirometry to identify patients with COPD [10]. To ensure the accuracy and reliability of the COPD diagnosis, the exclusion criteria were: (1) incomplete demographic data; (2) no pulmonary function test within one year before or after the diagnosis of COPD; and (3) COPD was not diagnosed after the lung function test. We also excluded those who died or were diagnosed with sepsis prior to being indexed.

The patients with sepsis were identified by ICD-9-CM codes for both an infectious process and acute organ dysfunction, which is similar to the method previously reported by Lai et al. [11]. For the patients with more than one episode of sepsis, only the first episode was included. The non-sepsis cohort was sampled as the reference group, and patients with a previous history of sepsis were excluded.

### 2.2. Demographic Characteristics and Comorbidities

The patients’ demographic characteristics including age, sex, monthly income (less than NT$ 19,100, NT$ 19,100-NT$ 41,999, and more than NT$ 42,000), hospital level at admission (medical center, regional, district, and others), number of severe exacerbations of COPD in the one year prior to the index date (never, 1, or ≥2 times/year)**,** and the index year of sepsis (2000–2011) were extracted. Underlying comorbid conditions were identified according to ICD-9-CM codes and grouped into the following categories: myocardial infarction, congestive heart failure, peripheral vascular disease, cerebrovascular disease, dementia, rheumatologic disease, peptic ulcer disease, hemiplegia or paraplegia, renal disease, diabetes, moderate/severe liver disease, and tumors. The Charlson Comorbidity Index (CCI) was used to determine the severity of the comorbidities in each patient. Important medications including aspirin, statins, non-steroidal anti-inflammatory drugs (NSAIDs), anti-hyperglycemic drugs, proton-pump inhibitors (PPIs), medications for hypertension and for COPD (long-acting beta agonist (LABAs), short-acting beta agonists (SABAs), long-acting muscarinic antagonists (LAMAs), and inhaled corticosteroids (ICSs)), were recorded.

### 2.3. Outcomes

The primary outcome was severe exacerbations of COPD, which was defined as a patient requiring hospital admission or an emergency department visit due to COPD. The secondary outcomes were pneumonia, serious pneumonia, and all-cause mortality. Pneumonia is identified according to ICD-9-CMcodes 480-486, and 507. Serious pneumonia was defined as pneumonia requiring invasive or non-invasive mechanical ventilation. Because of the high mortality rate and older-age in COPD patients, competing risk analysis using the Fine and Gray model was also performed [12]. All subjects were followed until the occurrence of events of interest, death or the end of the study (31 December 2011).

### 2.4. Statistical Analysis

Data analysis was performed using the SAS statistical package, version 9.4 (SAS Institute, Inc., Cary, NC, USA). The baseline characteristics are presented as descriptive statistics (means, standard deviations, counts, and percentages). Categorical baseline variables were compared using Pearson’s chi-square test or Fisher exact test. For continuous variable, the Kolmogorov-Smirnov test was applied to test for a normal distribution. When the continuous variable was normally distributed, an independent t-test was used. If the normality assumption was violated, a nonparametric Wilcoxon rank sum test was used. To minimize the imbalance in baseline characteristic covariates between the sepsis and non-sepsis groups, we performed 1:1 propensity score matching. Covariates that may have caused interference or biased the association between exposure and outcome of interest including demographic characteristics, comorbidities and severe exacerbations of COPD in the one year prior to the index date (never, 1, or ≥2 times/year) were included in the propensity matching.

The cumulative incidence rates of severe exacerbations were compared between the sepsis and non-sepsis groups before and after propensity score matching. Crude and adjusted hazard ratios (HRs) were calculated using Poisson regression with the non-sepsis cohort selected as the reference group.

The crude incidence rates of other outcomes (mortality, pneumonia and serious pneumonia) were obtained as the total number of events during the follow-up period divided by person-days. The Schoenfeld Residuals test was used to test the proportional hazards assumption. Cox proportional regression models were used with the non-sepsis cohort as the reference group. Crude HRs and adjusted HRs of individual outcomes were also evaluated after adjusting for the propensity score. We also used the Fine-Gray model in our incidence rate and time-to-event model to account for competing risks in the mortality model to derive subdistribution HRs (sub-HR) of secondary outcomes. A two-sided *p*-value < 0.05 was considered to indicate statistical significance in all analyses.

## 3. Results

### 3.1. Baseline Demographic Characteristics

**Figure 1:**
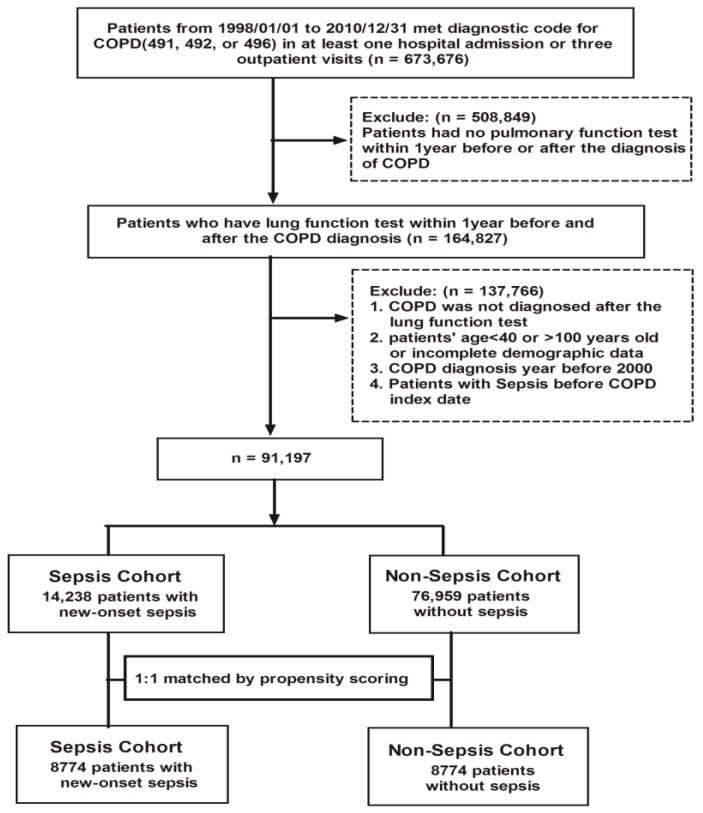
Flow chart of study cohort population selection. COPD: chronic obstructive pulmonary disease.

The medical records of 91,197 patients with COPD from 2000 to 2010 were reviewed, of whom 14,238 were enrolled as the sepsis cohort and 76,959 as the non-sepsis cohort. The demographic characteristics of both groups are summarized and shown in Table 1. The sepsis group tended to be older than the non-sepsis group. In addition, there were significant differences in sex, COPD index year, monthly income, medications for hypertension (beta-blockers, angiotensin-converting- enzyme inhibitors (ACEis) and angiotensin receptor blockers (ARBs), medications for COPD and statins between the two groups. In terms of baseline comorbidities, except for diabetes mellitus, the sepsis cohort had higher rates of myocardial infarction, congestive heart failure, peripheral vascular disease, cerebrovascular disease, dementia, rheumatologic disease, peptic ulcer disease, hemiplegia or paraplegia, renal disease, moderate or severe liver disease, and tumors. After 1:1 propensity score matching, 8774 patients with COPD were identified in each group to have similar characteristics including age, sex, income, baseline comorbidities and medications (Figure 1).

### 3.2. Outcomes Analysis 

Following matching, the post-index overall cumulative incidence rates of total acute exacerbations were 11.19/person-years in the sepsis group and 6.21/person-years in the non-sepsis group (adjusted HR = 1.38, 95% CI, 1.38–1.40). With regards to overall acute exacerbation events, the sepsis group also had higher risks of severe exacerbations (adjusted HR = 2.05, 95% CI, 2.02–2.08), severe exacerbations requiring hospitalization (adjusted HR = 2.30, 95% CI, 2.24–2.36), and severe exacerbations leading to an emergency room visit (adjusted HR = 1.91, 95% CI, 1.87–1.94). The results before matching were similar to those after matching (Table 2).

**Figure 2:**
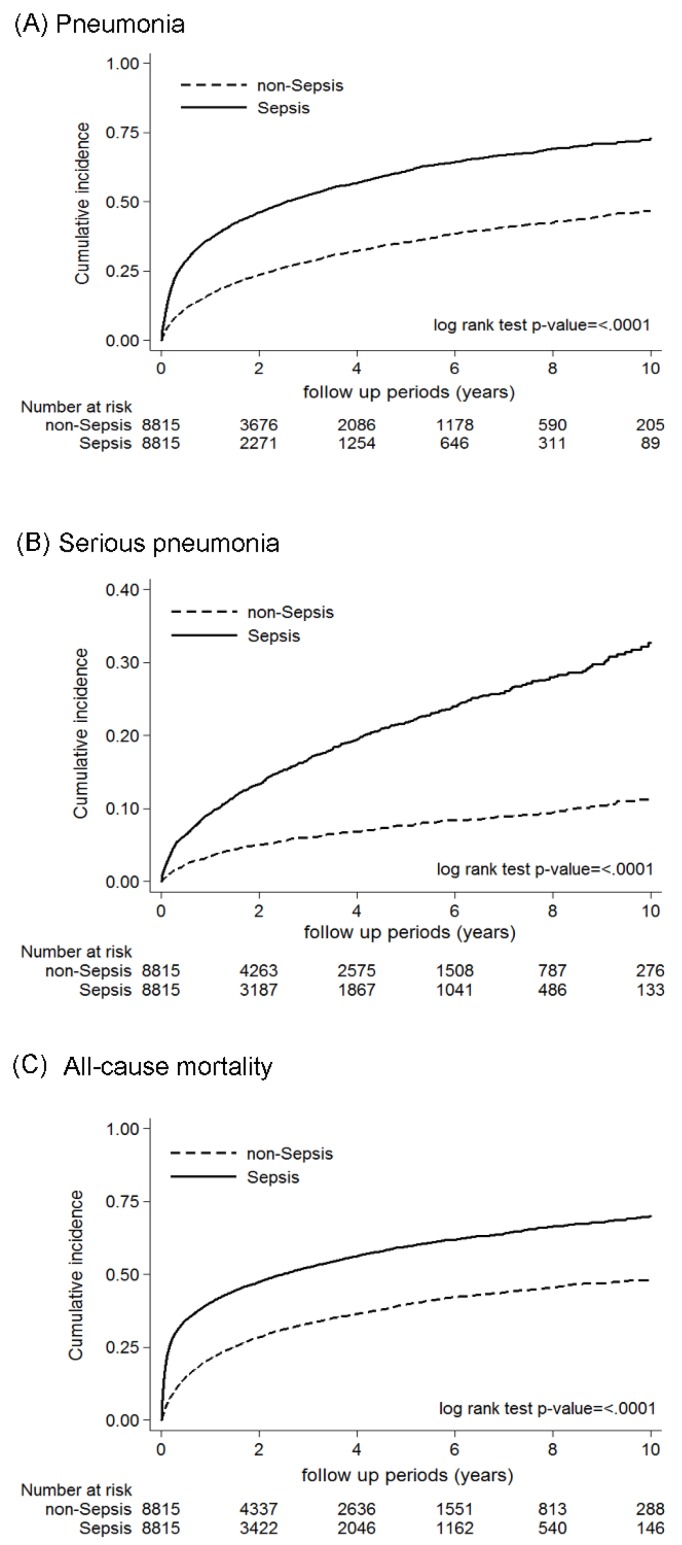
Kaplan-Meier analysis of the cumulative incidence for (**A**) pneumonia, (**B**) serious pneumonia and (**C**) mortality between the sepsis and non-sepsis cohort after matching.

Regarding the effect on secondary outcomes, the sepsis group had higher risks of mortality (incidence rate: 23.75 per 100 person-days vs. 11.34 per 100 person-days, adjusted HR = 2.27, 95% confidence interval (CI), 2.14–2.41), pneumonia (incidence rate: 26.41 per 100 person-days vs. 10.34 per 100 person-days, adjusted HR = 2.70, 95% CI, 2.5–2.91), and serious pneumonia (incidence rate: 5.84 per 100 person-days vs. 1.98 per 100 person-days, adjusted HR = 2.89, 95% CI, 2.5–3.33) after propensity score matching compared with the non-sepsis group. These results were consistent to those before propensity score matching. In competing risk analysis performed by adjusting for mortality due to causes other than the outcomes of interest, sepsis was still significantly associated with pneumonia (sub-HR = 1.86, 95% CI, 1.75–1.98) and serious pneumonia (sub-HR = 2.09, 95% CI, 1.86–2.35) after matching (Table 3). Kaplan-Meier analysis for the cumulative incidence also yielded significant differences and higher risks of mortality (*p* < 0.001), pneumonia (*p* < 0.001), and serious pneumonia (*p* < 0.001) in the sepsis group (Figure 2).

## 4. Discussion

This study compared two matched cohorts each with 8774 patients with COPD with or without sepsis, and found that the sepsis group had poorer outcomes due to higher risks of severe exacerbations, mortality, pneumonia, and serious pneumonia. After adjusting for mortality from causes other than the outcomes of interest as a competing risk and propensity score, the results still showed consistent findings. In addition, in subgroup analysis, the negative effect of sepsis on the outcomes of the patients with COPD remained significant. 

This study has several strengths. Spirometry is crucial and the most objective diagnostic tool used to diagnose COPD [13]. In order to ensure the accuracy of the diagnosis of COPD, we only enrolled patients who received a pulmonary function test within one year before and after the COPD diagnosis was made. Patients were also excluded if the COPD diagnosis was altered after the pulmonary test. The 2016 Surviving Sepsis Campaign (SSC) guidelines and the published 3rd International Consensus Definitions for sepsis and septic shock (Sepsis-3) revised definitions of sepsis emphasize organ dysfunction related to a dysregulated host response to infection [5,14]. Although the Sequential Organ Failure Assessment (SOFA) or quick SOFA (qSOFA) score is currently the most commonly used scoring system, the most appropriate scoring system to assess organ dysfunction is controversial [14,15,16]. In this study, we defined sepsis according to ICD-9-CM codes including all infections with related acute organ dysfunction as validated in a previous study [17]. These selection criteria were more comprehensive and similar to a modern definition of sepsis. We also performed propensity score matching to minimize the effects of possible confounding variables, and therefore we believe our findings are more representative and generalized.

Sepsis is associated with a high rate of mortality, however the reported rate varies widely from 10–52% depending on the how the data are collected [18,19,20]. Sepsis is also associated with poor long-term outcomes and increased risks of death following discharge, recurrent infections, readmission and worse quality of life. An increased incidence of bacterial infection of both respiratory tract and non-respiratory tract (i.e., urinary tract) origin has been reported in patients with COPD [6]. This increased incidence may be due to the following factors: 1. impaired barrier function of the respiratory tract in COPD resulting in bacterial colonization in the airway; 2. systemic inflammatory characteristic of COPD associated with multiple comorbidities [21]; 3. the usage of corticosteroids and anticholinergic agents [10,22,23,24]; and 4. possible adverse behavior, especially smoking [25]. However, to the best of our knowledge, the effect of sepsis on COPD subgroups has not been investigated in detail in previous studies.

Although the most common cause of acute exacerbations in patients with COPD appears to be from the respiratory tract infection (viral or bacterial) [13,26], exacerbations do not always coexist with an acute infection. The ecological features of infection, including increased bacterial burden with low community diversity associated with multiple indices of host inflammation such as alveolar neutrophilia and increased alveolar concentration of catecholamines, are consistently lacking in patients with exacerbations of COPD. However, critical illnesses such as sepsis will alter the ecosystem of the body’s microbiota due to various pathophysiological (i.e., glucose and electrolyte disturbances, impairment in IgA production, loss of secreted antimicrobial peptides in the mucosal barrier or endogenous opioid production) and therapeutic (sedatives, opiates, and catecholamines) factors, and is clearly involved in the pathogenesis of subsequent frequent exacerbations [27]. Furthermore, sepsis-induced organ dysfunction, such as renal function impairment, central nervous system dysfunction, and muscle wasting, may also contribute to long-term disabilities and increase the risk of mortality in patients with COPD [7,28,29].

Currently, the most commonly used tool to assess COPD is based on the “ABCD” grouping re-defined by the 2017 Global Initiative for Chronic Obstructive Pulmonary Disease (GOLD) guidelines, which also recommends the choice of therapy [13]. The COPD assessment tool takes account of the presence and severity of abnormalities in spirometry, clinical symptoms, exacerbation history and comorbidities, but not the influence of previous sepsis. In light of the fact that the impact of sepsis on patients with COPD is significant, sepsis should not be neglected when making treatment decisions for patients with COPD. Several therapies and approaches have been shown to have beneficial impacts on sepsis, including statins, theophylline, macrolides, and vaccinations, and they may play a role in treatment for COPD [30,31,32,33,34,35]. On the other hand, the agents commonly used to control COPD which may increase the risk of infection including corticosteroids and anticholinergics, should be used more cautiously in select patients [10,22,23]. Additional large and well-designed studies with more in-depth research on the control and prevention of sepsis in patients with COPD are essential and may lead to a more comprehensive COPD treatment strategy.

There are several limitations to this study. First, the diagnosis of COPD was made according to ICD-9 codes, which were mainly registered by clinical physicians. Although the accuracy was confirmed by strict screening criteria in our study, and the reliability of ICD-9 codes has been validated in previous studies [17,36,37], this issue remains a concern. Secondly, COPD is now considered to be a disease with single or combined “phenotypes” that describe the differences between individuals [38,39]. However, the impact of sepsis on different phenotypes of COPD was not available in this study. Third, the risk of mortality from sepsis varies depending on the severity of sepsis and comorbidities, however the association between the severity of sepsis and different groups of COPD (i.e., “ABCD” groups) could not be determined in the present study. Fourth, the exact results of spirometry were not be available in NHIRD. Thus, we could not classify the severity of COPD in this study. However, we used the number of COPD exacerbation episodes as an indicator and matched this data between sepsis and non-sepsis cohort. Fifth, our study revealed a relatively high proportion of patients received oral steroid therapy. Even though it was a real-world condition in Taiwan, this could limit the generalizability of our findings. Finally, similar to other database studies, some possible confounding factors such as smoking, environmental factors, appropriateness of antimicrobial use for pneumonia, Medical Research Council (MRC) Dyspnea scale, radiologic findings of pneumonia, and compliance of orders which may also have affected the outcomes were lacking.

## 5. Conclusion

Under Taiwan’s NHI program, our investigation showed that sepsis survivors among patients with COPD had a higher risk of severe exacerbations, pneumonia, serious pneumonia, and mortality compared to patients with COPD without sepsis. In light of these important findings, the negative effects of sepsis on patients with COPD should be considered in clinical practice. Furthermore, more efforts should be put into the early detection and prevention of sepsis to improve the prognosis and quality of life in patients with COPD.

## Figures and Tables

**Table 1:** Baseline characteristics of study population stratified by sepsis before and after propensity score matching.

	Before Propensity Score Matching (*n* = 91,197)			After Propensity Score Matching (*n* = 17,548)		
Patient characteristics	Non-Sepsis Cohort	Sepsis Cohort	*p*-Value	Non-Sepsis Cohort	Sepsis Cohort	*p*-Value
	*n* = 76,959	*n* = 14,238		*n* = 8774	*n* = 8774	
**Age (year)**	63.45 ± 11.56	70.7 ± 10.23	<0.0001 *	69.15 ± 10.88	68.93 ± 10.77	0.1994 *
**Years from COPD diagnosis to index date**	3.24 ± 2.83	4.13 ± 3.1	<0.0001 *	3.04 ± 3.04	3.2 ± 2.78	<0.0001 *
**Male sex**	52,530 (68.26%)	10,779 (75.71%)	<0.0001	6577 (74.96%)	6595 (75.17%)	0.7535
**Index year (COPD)**			<0.0001			0.9941
2000	12,013 (15.61%)	3547 (24.91%)		1746 (19.90%)	1708 (19.47%)	
2001	10,156 (13.20%)	2456 (17.25%)		1285 (14.65%)	1314 (14.98%)	
2002	8437 (10.96%)	1811 (12.72%)		1030 (11.74%)	1056 (12.04%)	
2003	6567 (8.53%)	1281 (9.00%)		777 (8.86%)	794 (9.05%)	
2004	6922 (8.99%)	1224 (8.60%)		768 (8.75%)	784 (8.94%)	
2005	5900 (7.67%)	1010 (7.09%)		682 (7.77%)	679 (7.74%)	
2006	5373 (6.98%)	811 (5.70%)		591 (6.74%)	582 (6.63%)	
2007	5512 (7.16%)	721 (5.06%)		594 (6.77%)	577 (6.58%)	
2008	5031 (6.54%)	560 (3.93%)		504 (5.74%)	492 (5.61%)	
2009	5485 (7.13%)	468 (3.29%)		435 (4.96%)	441 (5.03%)	
2010	5563 (7.23%)	349 (2.45%)		362 (4.13%)	347 (3.95%)	
**Monthly income, *n* (%)**			<0.0001			0.5955
<19,100	26,149 (33.98%)	5764 (40.48%)		3458 (39.41%)	3524 (40.16%)	
19,100–41,999	39,338 (51.12%)	7014 (49.26%)		4352 (49.60%)	4297 (48.97%)	
≥42,000	11,472 (14.91%)	1460 (10.25%)		964 (10.99%)	953 (10.86%)	
**Hospital level, *n* (%)**			<0.0001			0.0625
Level 1	15,271 (19.84%)	4986 (35.02%)		3097 (35.30%)	3137 (35.75%)	
Level 2	16,246 (21.11%)	6191 (43.48%)		3599 (41.02%)	3589 (40.90%)	
Level 3	9098 (11.82%)	2835 (19.91%)		1793 (20.44%)	1822 (20.77%)	
Level 4	36,344 (47.23%)	226 (1.59%)		285 (3.25%)	226 (2.58%)	
**COPD severe AE**			<0.0001			0.0184
0	61,315 (79.67%)	1466 (10.30%)		1381 (15.74%)	1466 (16.71%)	
1	7064 (9.18%)	3076 (21.60%)		2402 (27.38%)	2501 (28.50%)	
≥2	8580 (11.15%)	9696 (68.10%)		4991 (56.88%)	4807 (54.79%)	
***Medication for hypertension***						
Beta-blocker	23,006 (29.89%)	4752 (33.38%)	<0.0001	2963 (33.77%)	2959 (33.72%)	0.9491
ACEI or ARB	25,550 (33.20%)	6170 (43.33%)	<0.0001	3618 (41.24%)	3694 (42.10%)	0.2445
**Other medication**						
Aspirin	9537 (12.39%)	2474 (17.38%)	<0.0001	1433 (16.33%)	1436 (16.37%)	0.9512
Statin	10,515 (13.66%)	1625 (11.41%)	<0.0001	1069 (12.18%)	1059 (12.07%)	0.8171
NSAID	60,636 (78.79%)	11,275 (79.19%)	0.2836	7070 (80.58%)	7013 (79.93%)	0.2797
***COPD drug***						
Oral Steroid	29,962 (38.93%)	7986 (56.09%)	<0.0001	4795 (54.65%)	4765 (54.31%)	0.6493
LABA	865 (1.12%)	303 (2.13%)	<0.0001	213 (2.43%)	211 (2.40%)	0.9217
SABA	8354 (10.86%)	3149 (22.12%)	<0.0001	1817 (20.71%)	1808 (20.61%)	0.8667
LAMA	3716 (4.83%)	1273 (8.94%)	<0.0001	713 (8.13%)	691 (7.88%)	0.5404
ICS	12,567 (16.33%)	3749 (26.33%)	<0.0001	2218 (25.28%)	2225 (25.36%)	0.9033
**Baseline comorbidities**						
Charlson score	1.69 ± 1.13	2.35 ± 1.61	<0.0001 *	2.22 ± 1.58	2.21 ± 1.57	0.6532 *
Myocardial infarction	957 (1.24%)	432 (3.03%)	<0.0001	220 (2.51%)	223 (2.54%)	0.8852
Congestive heart failure	5133 (6.67%)	2660 (18.68%)	<0.0001	1370 (15.61%)	1347 (15.35%)	0.6313
Peripheral vascular disease	614 (0.80%)	228 (1.60%)	<0.0001	141 (1.61%)	129 (1.47%)	0.4617
Cerebrovascular disease	3895 (5.06%)	1550 (10.89%)	<0.0001	810 (9.23%)	805 (9.17%)	0.8961
Dementia	1075 (1.40%)	956 (6.71%)	<0.0001	325 (3.70%)	329 (3.75%)	0.8733
Reumatologic disease	688 (0.89%)	176 (1.24%)	0.0001	116 (1.32%)	112 (1.28%)	0.7897
Peptic ulcer disease	10,577 (13.74%)	2794 (19.62%)	<0.0001	1647 (18.77%)	1659 (18.91%)	0.8168
Hemiplegia or paraplegia	57 (0.07%)	29 (0.20%)	<0.0001	7 (0.08%)	7 (0.08%)	1
Renal disease	1966 (2.55%)	916 (6.43%)	<0.0001	426 (4.86%)	428 (4.88%)	0.9441
Diabetes	3407 (4.43%)	661 (4.64%)	0.2526	431 (4.91%)	421 (4.80%)	0.7254
Moderate or severe liver disease	3456 (4.49%)	1353 (9.50%)	<0.0001	824 (9.39%)	827 (9.43%)	0.9382
Tumor	9634 (12.52%)	2389 (16.78%)	<0.0001	1374 (15.66%)	1377 (15.69%)	0.9503

* Wilcoxon rank sum test. COPD: chronic obstructive pulmonary disease; ACEI: angiotensin-converting-enzyme inhibitor; ARB: angiotensin II receptor blocker; NSAID: nonsteroidal anti-inflammatory drugs; LABA: long acting beta agonist; SABA: short acting beta agonist; LAMA: long acting antimuscarinics; ICS: inhaled corticosteroid; AE: acute exacerbation.

**Table 2:** Cumulative incidence rates and hazard ratios of COPD patients with acute exacerbation, severe acute exacerbation, severe acute exacerbation requiring hospitalization and ED visit before and after propensity score matching in sepsis and non-sepsis cohort.

Outcomes	Sepsis Cohort	Non-Sepsis Cohort	Crude HR (95% CI)	Adjusted HR * (95% CI)
	IR (person-years)	IR (person-years)		
**Before propensity score matching**				
Total AE	11.93	4.67	1.51 (1.50–1.52)	1.48(1.48–1.49)
Severe AE	2.87	0.36	2.46 (2.42–2.50)	4.62(4.58–4.67)
Severe AE requiring hospitalization	1.17	0.13	2.76 (2.69–2.82)	5.44(5.36–5.53)
Severe AE requiring ED visit	1.70	0.24	2.30 (2.25–2.34)	4.19(4.14–4.24)
**After propensity score matching**				
Total AE	11.20	6.21	1.39 (1.38–1.40)	1.39(1.38–1.40)
Severe AE	2.44	0.92	2.05 (2.02–2.08)	2.05(2.02–2.08)
Severe AE requiring hospitalization	0.99	0.33	2.30 (2.24–2.36)	2.30(2.25–2.36)
Severe AE requiring ED visit	1.45	0.59	1.91 (1.87–1.94)	1.91(1.87–1.95)

*: adjusted for propensity score. AE: acute exacerbation; ED: emergent department; IR: incidence rate; HR: hazard ratio; CI: confidence interval.

**Table 3:** Incidence rates and risks of pneumonia, serious pneumonia of COPD patients comparing between sepsis and non-sepsis cohort before and after propensity score matching.

Outcomes	Non-Sepsis Cohort			Sepsis Cohort			Crude HR (95%CI)	Sub-HR *(95%CI)	Adjusted HR ^※^ (95%CI)
	Event	Person-Days	Incidence Rate (Event/Person Days)	Event	Person-Days	Incidence Rate (Event/Person Days)			
**Before propensity score matching**									
Mortality	11,229	260,716.08	4.31%	8479	27,981.77	30.30%	6.16 (5.99–6.34)		2.21 (2.12–2.3)
Serious pneumonia	1779	258,983.85	0.69%	1750	25,806.44	6.78%	8.87 (8.3–9.48)	2.18 (1.96–2.42)	3.76 (3.41–4.14)
Pneumonia	10,245	240,762.13	4.26%	5805	17,961.49	32.32%	6.37 (6.16–6.58)	1.74 (1.65–1.83)	2.62 (2.5–2.74)
**After propensity score matching**									
Mortality	2972	26,215.48	11.34%	4811	20,256.42	23.75%	2.28 (2.15–2.42)		2.27 (2.14–2.41)
Serious pneumonia	509	25,758.90	1.98%	1098	18,790.63	5.84%	2.88 (2.5–3.33)	2.09 (1.86–2.35)	2.89 (2.5–3.33)
Pneumonia	2280	22,044.38	10.34%	3554	13,455.45	26.41%	2.69 (2.49–2.9)	1.86 (1.75–1.98)	2.7 (2.5–2.91)

*: Adjusting for mortality as competing risk; ^※^: Adjusted for propensity score. HR, hazard ratio;. sub-HR, subdistribution hazard ratio.

## Abbreviations

TCORE	Taiwan Clinical Trial Consortium for Respiratory Diseases
COPD	Chronic Obstructive Pulmonary Disease
NHI	National Health Insurance
NHIRD	National Health Insurance Research Database
ICD-9-CM	International Classification of Diseases, Ninth Revision, Clinical Modification
ACCP/SCCM	American College of Chest Physicians/Society of Critical Care Medicine
CCI	Charlson comorbidity index
NSAID	Non-Steroidal Anti-inflammatory Drug
PPI	Proton-Pump Inhibitor
LABA	Long-Acting Beta Agonist
SABA	Short-Acting Beta Agonist
LAMA	Long-Acting Muscarinic Antagonist
ICS	Inhaled Corticosteroid
ACEi	Angiotensin-Converting-Enzyme inhibitors
ARB	Angiotensin Receptor Blocker
HR	Hazard ratios
sub-HR	subdistribution HRs
SSC	Surviving Sepsis Campaign
SOFA	Sequential Organ Failure Assessment
GOLD	Global Initiative for Chronic Obstructive Pulmonary Disease
